# Pedicle Morphometry of Subaxial Cervical Spine Using Computed Tomography Scans among Adult Ugandan Subpopulation

**DOI:** 10.1155/2022/6351465

**Published:** 2022-03-01

**Authors:** Ssebuggwawo Jonathan, Wani Muzeyi, Erem Geoffrey, Waiswa Gonzaga, Ssekitooleko Badru, Kajja Isaac

**Affiliations:** ^1^Department of Orthopaedic Surgery, School of Medicine, College of Health Sciences, Makerere University, Uganda; ^2^Clinical Epidemiology Unit, School of Medicine, College of Health Sciences, Makerere University, Uganda; ^3^Department of Radiology, School of Medicine, College of Health Sciences, Makerere University, Uganda; ^4^Department of General Surgery, School of Medicine, College of Health Sciences, Makerere University, Uganda

## Abstract

**Background:**

Accurate placement of pedicle screws in the subaxial cervical spine requires precise understanding of vertebra anatomy. Little is known about the morphometric characteristics of the subaxial cervical pedicle in the Ugandan population. The objective of the study was to determine the morphometric dimensions of pedicles in the subaxial cervical spine among the adult Ugandan population.

**Methods:**

We conducted a cross-sectional study from March to November 2019 among adult Ugandans with a normal cervical CT scan at Nsambya hospital in Kampala. Eligible participants were consecutively recruited into the study. Data on baseline characteristics and pedicle dimensions from the CT scan finding was collected using a structured questionnaire. Data was analysed using Stata 13.0. Pedicle dimensions for the different levels of subaxial cervical vertebrae were summarised as means and standard deviations, the Mann–Whitney test was used to compare pedicle dimensions for the different vertebra levels among females and males on both right and left sides, and the level of significance was set at 0.05.

**Results:**

A total of 700 subaxial cervical pedicles (C3-C7) from 49 males and 21 female participants were studied. Pedicle width diameter showed cephalocaudal gradual increment from C3 1.65(0.63) mm to 3.46(0.75) mm at C7. Pedicle height also showed an increase caudally with smallest diameter at C3 (1.98(0.76) mm) and largest at C5 in females (3.67(6.42) mm) and at C7 in males (3.83(0.76) mm). The pedicle height was wider than the pedicle width at all levels. The pedicle chord length gradually increased caudally in both sexes ranging from 29.08(1.35) mm at C3 to 32.53(3.19) mm at C7. The axial angles were oriented medially and showed no consistent trend ranging between 50° and 53°. The sagittal angles decreased as one moved from C3 to C7. The dimensions of females were significantly smaller than in males.

**Conclusion:**

Pedicle endosteal width was smaller than pedicle height dimensions at all levels. Pedicle cord length increased caudally. The pedicle dimensions, except angulations, were smaller in females than in males.

## 1. Introduction

There are a number of well researched and documented subaxial cervical spine fixation methods for different pathologies. These include but not limited to pedicle screws, lateral mass screws, interspinous wiring, laminar hooks, and plating. Among all these, pedicle screw fixation has demonstrated the best biomechanical attributes like a high pull-out strength. Among the five different human vertebra types (cervical, thoracic, lumbar, sacral, and coccygeal), the cervical spine exhibits the smallest and widest population variability in pedicle morphometry. This predisposes visceral structures contained in the cervical spine (the spinal cord, the nerve roots, and vertebral arteries) to damages during any form of instrumentation surgery [1–4]. Therefore, a thorough understanding of the osteology of the subaxial cervical spine is a prerequisite for safe surgery in this region. Studies of cervical morphometric dimensions have been done in different ethnic populations including Brazilians, Indians, Thais, Europeans, Chinese-Singaporeans, and Malaysians. Many indicated that there are interracial differences in the pedicle morphometric [5–9]. To date, no study has been conducted on any Ugandan racial groups to describe the osteological characteristics of their cervical spine pedicle.

A clear definition of the morphometric characteristic of the subaxial cervical spine improves choice of implants for spine surgery for any procedure but also aids the spine surgeons on the selection of appropriate pedicle screw sizes for the different demographic patient characteristics. Therefore, the objective of this study was to describe the morphometric dimensions of the subaxial cervical spine pedicles among adult Ugandans using computed tomography (CT) scans.

## 2. Methods

We conducted a cross-sectional study conducted at the radiology department of Nsambya hospital from 1^st^ March to 30^th^ November 2019. Nsambya hospital is a 361 bed private not for profit (PNFP) hospital located in Kampala city, Uganda. Its radiology department conducts up to 3 cervical spine CT scans weekly.

Use the formula of sample size for continuous variables, taking 95% level of confidence *D* = 0.2 mm and *S* = 0.83 mm.

A study by Chanplakorn et al. found the standard deviation of the PW to be 0.83 mm and taking *d* = 0.2 mm [10], and adding 5% to cater for nonresponse or incomplete data, we obtained a sample size of 70.

The study was conducted among Ugandans aged 18 years and above with normal CT scans of the cervical spine. These included images from patients who had presented to the radiology department either for cervical spine solely or entire vertebral spine CT scan depending on the indication. Cervical spine CT scans with evidence of fractures, dislocations, degenerative changes, infection, or neoplasia were excluded from this study. Informed consent for participation in the study was sought from participants whose images satisfied the selection criteria. CT scan images were done using a SOMATOM Perspective 128 slice CT scan machine (Siemens Healthineers, Germany). The CT scan machine had both helical and axial scanning modes with volumetric capabilities; reformatted images in sagittal and coronal planes for the entire cervical spine were required. This machine was a 2013 model, installed in January 2014 and was calibrated regularly.

## 3. Study Procedure

All included images were examined by two consultant radiologists, and in case of variations, the two radiologists met and resolved it. The demographic characteristics of patients, whose images were included, were noted.

Using the data extraction tool, the left and right pedicle morphometric dimensions for the subaxial spine for each participant were extracted from the reformatted images on the CT work station. Axial and sagittal cuts were made along the optimal pedicle axis to get the sagittal isthmus section using a software application on the CT scan work station called Digital Imaging and Communications in Medicine viewer software with a precision of 0.1 mm. The pedicle dimension data were measured and recorded on a data extraction form and then entered into Microsoft excel database. Data was then exported to Stata version 13.0 for analysis. Pedicle dimensions for the different levels of cervical vertebrae were summarised as means and standard deviations, the Mann–Whitney test was used to compare pedicle dimensions for the different vertebra levels among females and males on both the right and left sides, and the level of significance was set at 0.05. We also did correlations (Corr) between BMI and the pedicle dimensions.

The following pedicle morphometric measurements were taken: inner pedicle width: inner mediolateral diameter of the isthmus of the pedicle or width of cancellous core, inner pedicle height: inner super inferior diameter of the pedicle or the height of the cancellous core of the isthmus of the pedicle, pedicle transverse angle: angle between the sagittal plane and the longitudinal pedicle axis (LPA), pedicle sagittal angle: angle between the inferior vertebral endplate and LPA, and chord length (CL): distance from the pedicle entry point to the anterior aspect of the vertebral body. Measurements were carried twice for each dimension, and the average was noted.

## 4. Results

### 4.1. Description of Study Participants

A total of 70 participants were enrolled into the study, and 49 (%) were males. The age of the participants ranged from 19 to 76 years with a median age of 33.5 years, and the interquartile range was 20. The median BMI of participants was 25.6. A total of 700 subaxial cervical spine pedicles (C3-C7) were studied.

### 4.2. Axial Pedicle Dimensions

#### 4.2.1. Pedicle Width (PW)

The mean PW gradually increased from C3 to C7 as shown in Table 1. The overall PW ranged from 1.65 mm to 3.46 mm (Table 1). The smallest mean PW was found at C3 in both females (1.35 mm) and males (1.77 mm), while the largest mean PW was at C7 in both females (3.10 mm) and males (3.66 mm). The mean PW was smaller in females than in males at all levels (*p* < 0.05). Generally, there was no correction between PW and BMI; however, there was a weak correction of 0.18 at C3 and 0.15 at C7. The results are summarised in Table 1.

#### 4.2.2. Pedicle Chord Length

The overall mean chord length ranged from 29.75 mm to 31.99 mm (Table 2). The smallest mean chord length was found at C3 in both females (29.08 mm) and males (30.03 mm), while the largest mean valve was found at C7 in both females (30.73 mm) and males (32.53 mm). The mean chord length was smaller in females than in males at all levels (*p* < 0.05). The chord length increased as from C3 to C7. There was weak correlation between chord length and BMI. The results are summarised in Table 2.

#### 4.2.3. Pedicle Axial Angle

The overall mean axial angle (AA) ranged from 50.66° to 54.791° (Table 3). The smallest mean axial angle was found at C7 in females (49.1°) and at C3 in males (50.48°), while the largest mean axial angle was at C4 in both females (54.50°) and males (54.91°). There was no statistically significant difference between genders at any level (*p* > 0.05). There was no correlation between axial angles and the BMI except on the left of C3 (0.21), C4 (0.29), and C5 (0.21) where a very weak correlation was found. The results are summarised in Table 3.

### 4.3. Sagittal Pedicle Diameters

#### 4.3.1. Pedicle Height (PH)

The general mean PH ranged from 2.32 mm to 3.65 mm (Table 4). The smallest mean PH was found at C3 in both females (1.98 mm) and males (2.50 mm), while the largest mean PH was at C5 in females (3.67 mm) and at C7 in males (3.83 mm). The mean pH was smaller in females than in males at all levels (*p* < 0.05). There was a gradual increase in mean pH advancing caudally in the subaxial cervical spine. There was no correlation between pH and BMI at all levels. The results are summarised in Table 4 below.

#### 4.3.2. Pedicle Sagittal Angle (PSA)

The overall mean PSA ranged from 2.20° to 15.88° (Table 5). The smallest mean PSA was found at C7 in both females (1.62°) and males (2.45°). The largest mean PSA was found at C4 in both females (15.86°) and males (15.88°). There were no statistical differences among genders at all levels (*p* > 0.05). There was a weak negative correlation between PSA and BMI at C3 (0.36) on the right and C3 (0.16) on the left and at C6 (0.16) on the right and C6 (0.33) on the left. The results are summarised in Table 5 below.

## 5. Discussion

In this study, 70% of the participants were males which was in keeping with other similar studies that had a higher ratio of males to females [8, 11, 12] and this can be explained by the fact that most patients who required cervical CT scans were those involved in trauma of which males are more involved.

The pedicle parameters of females were found to be smaller than those of males, and the difference was statistically significant (*p* < 0.05). This was in keeping with the findings of the studies done locally for thoracic and lumbar spines as well as studies done in other populations such as Thailand and Caucasians [4, 10].This could be due to the fact that genetically females have a relatively smaller and shorter stature compared to male counter parts (Hill, 2017; Touraille & Gouyon, 2008).

### 5.1. Pedicle Endosteal/Inner Width

Pedicle endosteal width in this study was the smallest parameter of all parameters, and it continuously increased from C3 to C7, and this was in keeping with previous studies [4, 13, 14]. The pedicle width of the Ugandan population studied is smaller than that reported in studies elsewhere among Europeans and Americans as reported a systematic review study (Liu, Napolitano, & Ebraheim, 2010). In this study, the widest mean pedicle endosteal width was at C7 3.66 mm (0.62) in men and 3.10(1.05) in females. The mean endosteal pedicle width at C6 was 2.65(0.84) mm and 2.05(0.59) mm on the right in males and females, respectively, and 2.56(0.78) mm and 2.22(0.67) mm on the left in males and females, respectively, while at C7, it was 3.53(0.57) mm and 3.10(1.05) mm on the right in males and females, respectively, and 3.66(0.62) mm and 2.99(0.83) mm on the left in males and females, respectively.

Hence, it is only at C7 that 3.5 mm pedicle screws can be inserted bilaterally in both males and females because they have endosteal minimum diameter of more than 2.5 mm. At C6, it can be used in males on both right and left pedicles unlike in females and this is comparable to studies among Malaysians and Chinese [8, 14]. The reason for the small pedicle width among Ugandan population could be due to the difference in nutrition and environment as compared to other western population with larger pedicle width.

### 5.2. Pedicle Height

The endosteal pedicle height increased gradually caudally in both sexes with the smallest at C3 and largest at C5 in females and C7 in males, and this is not in agreement with the findings by Westerman et al. where the height reminded constant (Westermann et al., 2018), and this could be due the population difference. There was a significant difference between genders (*p* < 0.05), the females having smaller pedicle heights than male counterparts, and this is due to the fact that genetically males have a generally bigger and taller stature.

At all levels, the pedicle height was larger than the pedicle width and this trend was compared well with studies done elsewhere [10, 14–16]. This diameter should be taken into consideration during screw insertion with minimal risk of perforation or fracture of the superior and inferior pedicle cortices.

### 5.3. Chord Length

In this study, the mean overall chord length ranged from 29.75 mm to 31.99 mm and the values were comparable to other studies such as Herrero et al.'s study where it ranged from 29.4 mm to 33.4 mm, in an American study by Rao et al., which was 31.3 mm to 33.1 mm, in European by Leonard et al., which was 29.4 mm to 33.4 mm, and by Gupta et al., which was from 30.5 mm to 35.3 mm [4, 15, 17]. The length increased caudally from C3 to C7 with the smallest length at C3 29.75 mm and longest at C7 31.99 mm. However, this trend differs from Herrero et al. and Rao et al.'s studies which showed that the chord length was smallest at C7 level, and this difference is possibly due to the fact that the populations studied are different from our population [6, 17].

The chord length was longer in men than in female counterparts, and the differences was statistically significant (*p* < 0.05) at all levels which was in agreement in other studies (Rao et al., 2008). This is because females genetically have a shorter and shorter stature compared to the male counterparts [18, 19]. Chord length is an important factor in pullout strength of a pedicle screw; hence, proper assessment of a screw length is required [20, 21].

### 5.4. Angulation

In this study, findings demonstrate that the pedicle screw should be a little more directed medially ranging from 50.29° to 54.79° and is dependent on the spine level. Pedicles in the current study are directed more medially as compared to the previous studies conducted on Chinese, European, and American populations [14, 22]. Sagittal angulation showed cranial orientation in the upper segment of subaxial cervical spine; however, as we moved caudally from C3 to C7, the orientation became horizontal to the superior end plate ranging from 15.37° to 2.66°.

There was no statistically significant difference among males and females in transverse angle and the pedicle sagittal angle at all levels of the subaxial cervical spine as it also found in other studies [10, 11].

There were large individual variations in our study population as evidenced by the relatively wide PTA range and large standard deviation for each cervical vertebra level; hence, preoperative CT scan evaluation of PTA is crucial to determine the safe and correct angle for pedicle screw placement. Standard angles for screw insertion should not be recommended in our population due to the variations and unforgiving anatomic boundaries of the subaxial cervical spine.

## 6. Conclusions and Recommendations

### 6.1. Conclusions

The overall pedicle endosteal width is less than 4 mm at all levels in both genders, and it increases from C3 to C7.

Pedicle endosteal width is smaller than pedicle height dimensions at all levels; hence, it is the primary dimension used to determine the screw diameter, and it increases caudally.

The pedicles of the upper subaxial cervical spine are oriented cranially and then become horizontal in the lower subaxial cervical pedicles, and they are oriented medially to the midline.

Pedicle cord length increases caudally, and it is the determinant for the screw length.

There is no correlation between pedicle dimensions and BMI in the study population.

The pedicle dimensions are smaller in females than in males except for the angulations which show no gender difference among the population.

### 6.2. Recommendations

A 4.5 mm and 4 mm pedicle screw diameter is not safe to be used in the subaxial cervical spine transpedicular fixation among Ugandan populations due the smaller pedicle width. Hence, advise the different spine implant designers and manufacturers to customize cervical spine pedicle screws to Uganda's population due to the smaller sizes of our dimensions as compared to other populations.

The pedicle screw length ranging from 29 mm to 32 mm and axial angulation of 50° from the midline is appropriate in our population. Transpedicular screw insertion should be avoided in higher subaxial cervical pedicles because such attempt can be detrimental in Uganda's population because of the smaller pedicle dimensions.

A large study in different parts of the country should be carried to be able to apply these findings to the whole Ugandan population.

## Figures and Tables

**Table 1:** Showing pedicle width of 70 participants.

	PW(SD)	*p* value	Corr		*p* value	Corr
	Right			Left		
C3						
Overall	1.65(0.63)	0.008	0.18	1.75(0.63)	0.005	-0.04
Male	1.77(0.60)			1.88(0.57)		
Female	1.35(0.60)			1.44(0.66)		
C4						
Overall	1.82(0.77)	0.003	0.07	1.75(0.64)	0.042	0.14
Male	2.01(0.73)			1.84(0.62)		
Female	1.37(0.70)			1.52(0.62)		
C5						
Overall	2.13(0.73)	0.013	0.06	2.06(0.66)	0.027	0.14
Male	2.27(0.73)			2.18(0.66)		
Female	1.81(0.64)			1.78(0.59)		
C6						
Overall	2.47(0.82)	0.006	0.009	2.46(0.77)	0.11	0.04
Male	2.65(0.84)			2.56(0.78)		
Female	2.05(0.59)			2.22(0.67)		
C7						
Overall	3.41(0.76)	0.021	0.15	3.46(0.75)	0.002	-0.08
Male	3.53(0.57)			3.66(0.62)		
Female	3.10(1.05)			2.99(0.83)		

PW: pedicle width (SD); Corr: correlation coefficient of pedicle parameter with BM.

**Table 2:** Cord length (cl) of 70 participants.

	CL	*p* value	Corr		*p* value	Corr
	Right			Left		
C3						
Overall	30.01(1.75)	0.003	0.28	29.75(1.64)	0.02	0.09
Male	30.41(1.74)			30.03(1.68)		
Female	29.09(1.40)			29.08(1.35)		
C4						
Overall	30.15(1.48)	0.001	0.26	30.31(1.74)	0.002	0.08
Male	30.49(1.32)			30.74(1.55)		
Female	29.35(1.58)			29.30(1.75)		
C5						
Overall	30.84(1.91)	0.002	0.2	30.79(1.92)	0.003	0.07
Male	31.39(1.74)			31.19(1.75)		
Female	29.55(1.70)			29.86(2.02)		
C6						
Overall	31.85(1.89)	<0.001	0.08	31.89(1.95)	0.0002	0.07
Male	32.44(1.65)			32.40(1.86)		
Female	30.46(1.70)			30.67(1.60)		
C7						
Overall	31.99(2.88)	0.0001	0.1	31.99(2.18)	0.0003	0.04
Male	32.53(3.19)			32.52(2.13)		
Female	30.73(1.33)			30.78(1.83)		

CL: chord length (SD).

**Table 3:** Axial cervical pedicle angles of 70 participants.

	AA	*p* value	Corr		*p* value	Corr
	Right			Left		
C3						
Overall	50.72(3.89)	0.457	0.11	50.82(4.63)	0.608	0.21
Male	50.48(3.90)			51.67(5.10)		
Female	51.29(3.92)			52.17(3.35)		
C4						
Overall	52.47(3.58)	0.934	0.09	54.79(3.09)	0.546	0.29
Male	52.51(3.83)			54.91(3.22)		
Female	52.38(2.99)			54.50(2.83)		
C5						
Overall	52.32(3.65)	0.008	0.02	52.96(3.95)	0.204	0.21
Male	53.04(3.57)			53.35(4.45)		
Female	50.64(3.33)			52.05(2.26)		
C6						
Overall	50.89(4.03)	0.422	0.04	51.11(3.99)	0.013	0.14
Male	51.20(3.79)			51.83(3.77)		
Female	50.14(4.55)			49.45(4.07)		
C7						
Overall	50.66(3.52)	0.48	0.07	50.29(3.42)	0.096	-0.08
Male	50.86(3.34)			50.80(3.10)		
Female	50.29(3.96)			49.12(3.89)		

AA: axial angle (SD).

**Table 4:** Pedicle height of 70 participants.

	PH	*p* value	Corr		*p* value	Corr
	Right			Left		
C3						
Overall	2.37(0.66)	0.012	0.06	2.32(0.75)	0.023	-0.04
Male	2.51(0.59)			2.50(0.69)		
Female	2.02(0.69)			1.98(0.76)		
C4						
Overall	2.54(0.78)	0.001	0.03	2.53(0.83)	0.06	0.21
Male	2.75(0.66)			2.66(0.82)		
Female	2.06(0.84)			2.23(0.79)		
C5						
Overall	2.60(0.66)	0.047	0.07	3.05(3.51)	0.06	-0.03
Male	2.72(0.61)			2.79(0.56)		
Female	2.32(0.69)			3.67(6.42)		
C6						
Overall	2.85(0.71)	0.004	0.09	2.90(0.74)	0.028	0.15
Male	3.02(0.60)			3.03(0.62)		
Female	2.43(0.77)			2.60(0.90)		
C7						
Overall	3.65(0.80)	0.005	0.09	3.63(0.84)	0.127	0.01
Male	3.83(0.76)			3.77(0.73)		
Female	3.24(0.76)			3.31(0.99)		

PH: pedicle height (SD).

**Table 5:** Pedicle sagittal cervical angles of pedicles of 70 participants.

	PSA	*p* value	Corr		*p* value	Corr
	Right			Left		
C3						
Overall	11.62(4.52)	0.949	-0.36	15.37(7.93)	0.882	-0.16
Male	11.51(4,24)			15.81(8.9)		
Female	11.88(5.22)			14.38(4.81)		
C4						
Overall	9.58(4.43)	0.913	0.05	15.88(5.15)	0.913	0.1
Male	9.59(4.14)			15.88(6.45)		
Female	9.57(5.14)			15.86(8.19)		
C5						
Overall	5.79(4.03)	0.422	-0.2	6.53(6.79)	0.546	-0.15
Male	6.12(4.35)			5.69(3.98)		
Female	5.02(3.13)			8.50(10.73)		
C6						
Overall	3.03(2.76)	0.331	-0.16	3.76(2.99)	0.657	-0.33
Male	3.31(2.99)			3.96(3.22)		
Female	2.40(2.04)			3.29(2.33)		
C7						
Overall	2.20(2.81)	0.24	-0.06	2.66(3.13)	0.785	-0.1
Male	2.45(2.98)			2.73(3.39)		
Female	1.62(2.34)			2.50(2.46)		

PSA: pedicle sagittal angle (SD).

## Data Availability

The data for this study is available on request, and interested researchers may submit queries related to data access to SOMREC (rresearch9@gmail.com) or corresponding author (jssebuggwawo@gmail.com).
